# A Case of Spontaneous Transdiaphragmatic Intercostal Hernia with Contralateral Injury, and Review of the Literature

**DOI:** 10.1155/2017/7416092

**Published:** 2017-02-23

**Authors:** Alexander A. Chapman, Steven B. Duff

**Affiliations:** ^1^Department of General Surgery, Riverside Methodist Hospital, Columbus, OH 43214, USA; ^2^OhioHealth Heart, Lung and Vascular Surgeons, Riverside Methodist Hospital, Columbus, OH 43214, USA

## Abstract

This case report discusses the diagnosis and management of a 67-year-old male presenting with a spontaneous transdiaphragmatic intercostal hernia with contralateral intercostal hernia. The patient had a history of chronic obstructive pulmonary disease (COPD) exacerbations requiring multiple prolonged courses of steroids. The patient was ultimately diagnosed with computed tomography (CT) and underwent surgical repair via thoracotomy with primary repair of the diaphragmatic defect. The patient's postoperative course was uncomplicated. A review of the literature since the first similar case in 1977 recognizes the propensity of this injury to be found in patients with COPD and chronic steroid usage, as well as its diagnosis and management. The case reviewed is the second documented case of a concurrent abdominal wall herniation and the first one with a contralateral injury. It is important for clinicians to be aware of this pathology when evaluating patients with COPD and chronic steroid usage.

## 1. Introduction

The authors report a rare case of combined cough-induced transdiaphragmatic and abdominal intercostal hernia with associated rib fractures, visceral herniation, and contralateral abdominal intercostal hernia, as well as the management of this condition, and a review of the literature to date. These findings are typically the result of blunt trauma; it has been rarely appreciated in the literature in atraumatic patients following episodes of severe coughing or exertion, as is the case in this report [1].

## 2. Case Report

A 67-year-old male was referred to the authors after undergoing chest X-ray (CXR) with evidence of left-sided rib fracture and pleural effusion on 27 January 2015. The patient had developed an intractable cough in December and was started on prednisone and antibiotics by a pulmonologist. The patient was admitted with COPD exacerbations twice in January for intravenous steroid and antibiotic therapy. Following the second time of hospitalization, the patient had some improvement and was discharged on a steroid taper.

**Figure 1:**
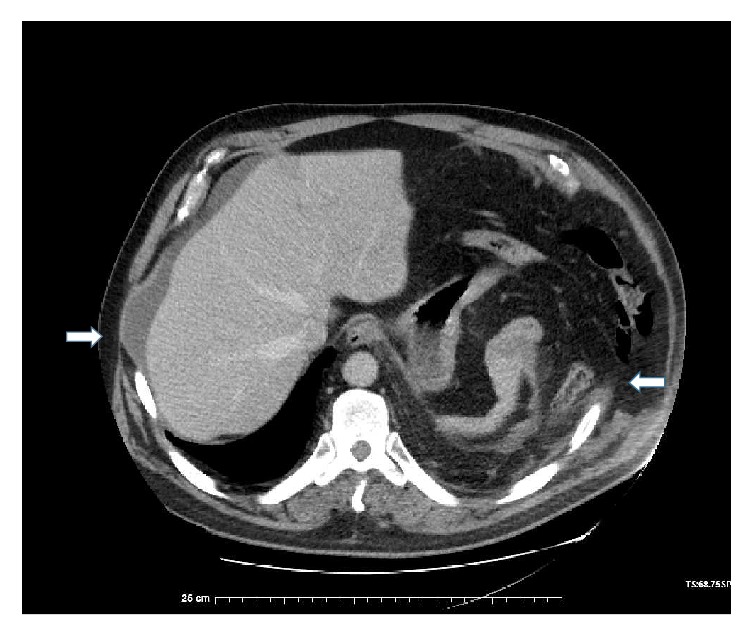


**Figure 2:**
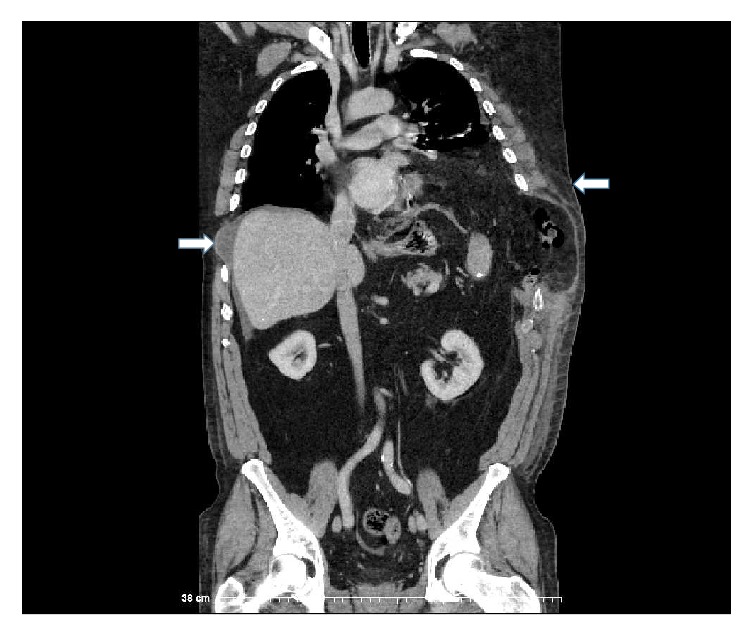


Three days following discharge, CXR and dedicated left-sided rib films showed a displaced left 8th rib fracture and left-sided effusion. Physical examination was notable for a 10 × 15 cm mass of the left chest flank associated with ecchymosis. The patient was also seen to have paradoxical motion of this mass with respiration. When lying in the right lateral decubitus position, there was note of concavity at the left 8th rib space. The patient denied respiratory distress but did exhibit decreased breath sounds on the left side. A CT was obtained with evidence of rupture of the left hemidiaphragm measuring 11.4 cm with herniation of omentum, splenic flexure, stomach, and anterior spleen into the chest cavity. There was displacement of bilateral 7th and 8th ribs with a left-sided 8th rib fracture and herniation of the abdominal contents through the intercostal space (Figures 1 and 2). This herniation transcended the diaphragmatic injury and involved a subdiaphragmatic abdominal intercostal hernia on the left side. An abdominal intercostal herniation was also evident on the right side in the space created by the 7th and 8th rib displacement.

Given the complex nature of the injury, surgical repair of the diaphragm and hernia was required. The patient underwent a left posterolateral thoracotomy; the incision was made over the defect in the chest wall. Omentum, transverse colon, and stomach were encountered in the extrathoracic hernia sac. These structures were mobilized into the peritoneum; there was no evidence of ischemia and therefore no resection was required; the hernia sac was excised. The diaphragmatic rupture was repaired with 2-0 pledgeted prolene. Immediately following the repair, the patient had a severe coughing episode intraoperatively resulting in tearing of the medial portion of the repair. Because there was not enough healthy diaphragm to repair the new defect primarily, a dual mesh Gore-Tex patch and 2-0 pledgeted prolene were used to close the defect. The left lower lobe was then reinflated and the intercostal defect was repaired with heavy vicryl figure-of-eight pericostal sutures. Two chest tubes were left in place.

The patient recovered without further incident; a steroid taper and antibiotics were continued upon discharge. The authors have no plans for repair of the right-sided defect as it is asymptomatic and without herniation of bowel. The patient has been seen in follow-up through December of 2016, nearly two years after initial presentation. At that time, the patient had no evidence of recurrence and was exercising regularly without exertional dyspnea or chest pain.

## 3. Discussion

An injury in which the abdominal viscera herniate through the diaphragm into the thoracic cavity and through the chest wall is known as a transdiaphragmatic intercostal hernia (TDIH). This requires two distinct defects and is often associated with rib fractures at the intercostal hernia [1, 2]. TDIH is an uncommon phenomenon that results typically from a traumatic etiology [3]. TDIH has also been noted in the literature to occur spontaneously due to episodes of increased pressure gradient between the intraperitoneal and intrathoracic cavities, such as emesis, straining, defecation, and coughing [4]. There is documentation of injuries from severe cough including rib fractures, pneumothorax [5], diaphragmatic rupture, abdominal intercostal hernias [6], diaphragmatic hernias, and rarely all of those aforementioned [1]. Of note, the authors were able to directly visualize this pathology intraoperatively: just following repair, the patient had a violent cough that tore the medial portion of the diaphragm, requiring placement of a mesh.

One of the mechanisms behind rib fractures associated with violent coughing is postulated to be similar to a stress fracture in that the force applied from the cough exceeds the elasticity of the bone. Another thought is that the fracture is associated with the opposing forces of the intercostal, diaphragmatic, abdominal, and serratus muscles on the rib [5]. This explains the reason these fractures are often found laterally and between the 5th and 9th ribs [7].

TDIH is typically noted initially on CXR; ultrasonography, magnetic resonance imaging (MRI), and direct visualization have also been used to make the diagnosis. The best modality, however, appears to be CT [8, 9]. TDIH has been documented to occur acutely [4], over several months [10], or over the course of years [9]. The nature of the chronic presentation is thought to be attributed to the persistent gradient between the intraperitoneal and intrathoracic cavities, as well as the continuous motion of the diaphragm causing slow expansion of the defect and prevention of healing [11].

It has been postulated that, given the high incidence of COPD in these patients, they are often on long term or frequent doses of steroids, as was the patient presented. Concomitant use of steroids was also documented by Rogers et al. [2] and Kallay et al. [4]. This raises the question as to whether the detrimental effects of steroids on healing predispose patients to diaphragmatic injuries, specifically regarding small defects caused by the constant shear and stress in this region [1, 4]. Lasithiotakis et al. [12] describe a case in which a patient presented with a spontaneous incarcerated TDIH. This was attributed to the wearing of a tight “milking belt” in a patient that denied even severe coughing, straining, or emesis [2]. The etiology was thought to be due to an increase in intraperitoneal pressure due to the belt, causing eventual thinning and atrophy of the diaphragm and intercostal muscles. This again supports the suspicion of suboptimal healing in these regions predisposing patients to this pathology.

A review of the literature revealed a total of 14 cases of TDIH, the oldest dating back to 1977. These cases as well as the one presented are reviewed in Table 1 [13–15]. Ten of the 15 cases had associated rib fractures, all of which involved ribs between 7 and 9. This again supports the explanation of cough-associated rib fractures described above. Regarding laterality, seven of the 15 were right-sided, six were left-sided, and one was unclear. The case presented above was the only bilateral injury in the literature reviewed (the contralateral injury being a spontaneous intercostal hernia with rib displacement). All of the patients were older than 50, and only one of the patients was female. Cough or pathology inducing episodes of coughing were comorbidities in most of the patients presenting with TDIH. There were only two reports of concurrent ipsilateral abdominal wall hernias traversing the diaphragm associated with TDIH: the one presented above and that of Abu-Gazala et al. [10]. Both of these patients were males with rib fractures, though the injury occurred on different sides.

Based on the literature review, it seems that the most common symptoms associated with spontaneous TDIH are pain, protrusion/swelling, and ecchymosis. An important aspect in the physical exam is that when the patient lies in the lateral decubitus position on the side contralateral to the injury, there is a concave defect noted at the hernia site, often with paradoxical motion [12]. This pathology, though rare, requires a high index of suspicion. Physicians should consider this diagnosis in patients presenting with the aforementioned symptoms especially following an episode of substantial exertion [5]. Suspicion is best confirmed with CT [9, 12].

Definitive management of a TDIH is primarily surgical. In the authors' review, all cases required surgical repair except for one which is pending optimization, though definitive management is planned. Repair typically consists of a thoracotomy over the defect; however, an abdominal approach has been utilized in two of the cases, and a thoracoabdominal approach has been utilized in three cases. The hernia defect is reduced with excision of the hernia sac and repair of the diaphragmatic defect. This can be done primarily or with mesh, as in the case presented. Finally, the intercostal defect is reapproximated and the incision was closed. Evidence of ischemia or necrosis mandates excision of that portion of bowel. Chest tube drainage of the thorax is also employed [5, 7, 9, 12].

## 4. Conclusion

Spontaneous TDIH is a rare complication of sudden changes in diaphragmatic-peritoneal pressure gradient. There are currently 15 cases reported in literature, this case being the second with a combined thoracic and abdominal component and the first with a contralateral injury as well. This injury can quickly progress given the nature of the constant force of the diaphragmatic, intercostal, and abdominal muscles, which can lead to strangulation. It is important for clinicians to have a high index of suspicion for this etiology, particularly in patients presenting with ecchymosis and a painful bulge in their chest following severe coughing or straining. Patients with suspicion for this pathology should undergo CT for diagnosis and should be definitively managed with surgical repair.

## Figures and Tables

**Table 1:** Characteristics of reviewed reports of transdiaphragmatic intercostal hernia.

	Age	Sex	Underlying disease process	Rib fractured	Laterality	Presence of abdominal wall hernia	Steroid usage	Symptoms	Surgical approach
Croce and Mehta, 1979	74	M	COPD	9	Right	No	N/A	Pain, protrusion, ecchymosis	Thoracoabdominal
Cole et al., 1986	72	M	COPD	8, 9, 10	Left	No	N/A	Pain, protrusion, ecchymosis	Thoracotomy
Rogers et al., 1996	64	M	Sarcoid	9	Left	No	Yes	Pain, protrusion, ecchymosis	Thoracotomy
Kallay et al., 2000	63	F	Asthma	None	Left	No	Yes	Pain, ecchymosis, dyspnea	Abdominal
Khan et al., 2006	75	M	COPD	None	Right	No	N/A	Pain, swelling	Thoracotomy
Chaar et al., 2007	54	M	COPD	7	Left	No	N/A	Pain, swelling	Thoracoabdominal
Daniel et al., 2008	70	M	N/A	None	Left	No	N/A	Pain, dyspnea	Thoracotomy
Aggarwal et al., 2010	65	M	Remote PNA	None	Left	No	N/A	Swelling, dyspnea	Thoracoabdominal
Abu-Gazala et al., 2011	59	M	Cough	9	Right	Yes	N/A	Pain, protrusion, ecchymosis	Thoracotomy
Lasithiotakis et al., 2011	78	M	Tight belt	None	Left	No	N/A	Pain, protrusion, ecchymosis	Abdominal
Macedo et al., 2012	53	M	PNA	8	Right	No	N/A	Pain, protrusion, ecchymosis	Thoracotomy
Macedo et al., 2012	71	M	Cough	8	Right	No	N/A	Pain, protrusion, ecchymosis	Thoracotomy
Macedo et al., 2012	64	M	COPD	None	Right	No	N/A	Pain, protrusion, ecchymosis	Thoracotomy
Sit et al., 2015	81	M	COPD	7, 8, 9	Right	No	N/A	Pain, protrusion, ecchymosis	Pending repair
Chapman et al., 2015	66	M	Asthma	8	Bilateral	Yes	Yes	Pain, protrusion, ecchymosis	Thoracotomy

## Abbreviations

CXR: Chest X-ray
COPD: Chronic obstructive pulmonary disease
CT: Computed tomography
MRI: Magnetic resonance imaging
TDIH: Transdiaphragmatic intercostal hernia.

## References

[1] Chaar C. I. O., Attanasio P., Detterbeck F. (2008). Disruption of the costal margin with transdiaphragmatic abdominal herniation induced by coughing. *American Surgeon*.

[2] Rogers F. B., Leavitt B. J., Jensen P. E. (1996). Traumatic transdiaphragmatic intercostal hernia secondary to coughing: case report and review of the literature. *Journal of Trauma and Acute Care Surgery*.

[3] Sharma O. P. (2001). Transdiaphragmatic intercostal hernia: review of the world literature. *Journal of Trauma*.

[4] Kallay N., Crim L., Dunagan D. P., Kavanagh P. V., Meredith W., Haponik E. F. (2000). Massive left diaphragmatic separation and rupture due to coughing during an asthma exacerbation. *Southern Medical Journal*.

[5] Daniel R., Naidu B., Khalil-Marzouk J. (2008). Cough-induced rib fracture and diaphragmatic rupture resulting in simultaneous abdominal visceral herniation into the left hemithorax and subcutaneously. *European Journal of Cardio-thoracic Surgery*.

[6] Connery A., Mutvalli E. (2010). Cough-induced abdominal intercostal hernia. *JRSM Short Reports*.

[7] Sit A., Chatterjee S., Das I. (2016). A case of spontaneous transdiaphragmatic intercostal hernia of bowel loops and omentum with herniation of lung—a very rare entity. *Egyptian Journal of Chest Diseases and Tuberculosis*.

[8] Kurer M. A., Bradford I. M. J. (2006). Laparoscopic repair of abdominal intercostal hernia: a case report and review of the literature. *Surgical Laparoscopy, Endoscopy and Percutaneous Techniques*.

[9] Macedo A. C. S., Kay F. U., Terra R. M., De Campos J. R. M., Aranha A. G. A., Funari M. B. D. G. (2013). Transdiaphragmatic intercostal hernia: imaging aspects in three cases. *Jornal Brasileiro de Pneumologia*.

[10] Abu-Gazala M., Ratnayake A., Abu-Gazala S., Bala M. (2013). An enigma of spontaneous combined transdiaphragmatic, intercostal and abdominal wall hernia. *Hernia*.

[11] Aggarwal G., Khandelwal G., Shukla S., Maheshwari A., Mathur R., Acharya D. (2012). Spontaneous transdiaphragmatic intercostal hernia: a rare clinical entity. *Hernia*.

[12] Lasithiotakis K., Venianaki M., Tsavalas N. (2011). Incarcerated spontaneous transdiaphragmatic intercostal hernia. *International Journal of Surgery Case Reports*.

[13] Croce E. J., Mehta V. A. (1979). Intercostal pleuroperitoneal hernia. *Journal of Thoracic and Cardiovascular Surgery*.

[14] Cole F. H., Miller M. P., Jones C. V. (1986). Transdiaphragmatic intercostal hernia. *Annals of Thoracic Surgery*.

[15] Khan A. S., Bakhshi G. D., Khan A. A., Kerkar P. B., Chavan P. R., Sarangi S. (2006). Transdiaphragmatic intercostal hernia due to chronic cough. *Indian Journal of Gastroenterology*.

